# Accuracy of Chest Computed Tomography in Distinguishing Cystic Pleuropulmonary Blastoma From Benign Congenital Lung Malformations in Children

**DOI:** 10.1001/jamanetworkopen.2022.19814

**Published:** 2022-06-30

**Authors:** Abigail J. Engwall-Gill, Sherwin S. Chan, Kevin P. Boyd, Jacqueline M. Saito, Mary E. Fallat, Shawn D. St Peter, Stephanie Bolger-Theut, Eric J. Crotty, Jared R. Green, Rebecca L. Hulett Bowling, Sachin S. Kumbhar, Mantosh S. Rattan, Cody M. Young, Joseph K. Canner, Katherine J. Deans, Samir K. Gadepalli, Michael A. Helmrath, Ronald B. Hirschl, Rashmi Kabre, Dave R. Lal, Matthew P. Landman, Charles M. Leys, Grace Z. Mak, Peter C. Minneci, Tiffany N. Wright, Shaun M. Kunisaki

**Affiliations:** 1 Division of General Pediatric Surgery, Johns Hopkins Children’s Center, Johns Hopkins University School of Medicine, Baltimore, Maryland; 2 Department of Pediatric Radiology, Children’s Mercy Hospital, University of Missouri–Kansas City School of Medicine, Kansas City; 3 Department of Pediatric Radiology, Children’s Wisconsin, Medical College of Wisconsin, Milwaukee; 4 Department of Pediatric Surgery, St Louis Children’s Hospital, Washington University School of Medicine in St Louis, St Louis, Missouri; 5 Division of Pediatric Surgery, Norton Children’s Hospital, University of Louisville, Louisville, Kentucky; 6 Division of Pediatric Surgery, Children’s Mercy Hospital, University of Missouri–Kansas City School of Medicine, Kansas City; 7 Department of Pediatric Radiology, Cincinnati Children’s Hospital Medical Center, University of Cincinnati, Cincinnati, Ohio; 8 Department of Pediatric Radiology, Ann & Robert H. Lurie Children’s Hospital of Chicago, Northwestern University Feinberg School of Medicine, Chicago, Illinois; 9 Department of Pediatric Radiology, St Louis Children’s Hospital, Washington University School of Medicine in St Louis, St Louis, St Louis, Missouri; 10 Department of Pediatric Radiology, Nationwide Children’s Hospital, The Ohio State University College of Medicine, Columbus; 11 Center for Surgery Outcomes Research, Johns Hopkins University School of Medicine, Baltimore, Maryland; 12 Center for Surgical Outcomes Research, Abigail Wexner Research Institute, Department of Surgery, Nationwide Children’s Hospital, The Ohio State University College of Medicine, Columbus; 13 Section of Pediatric Surgery, C. S. Mott Children’s and Von Voigtlander Women’s Hospital, University of Michigan Medical School, Ann Arbor; 14 Division of Pediatric Surgery, Cincinnati Children’s Hospital Medical Center, University of Cincinnati, Cincinnati, Ohio; 15 Division of Pediatric Surgery, Ann & Robert H. Lurie Children’s Hospital of Chicago, Northwestern University Feinberg School of Medicine, Chicago, Illinois; 16 Division of Pediatric Surgery, Children’s Wisconsin, Medical College of Wisconsin, Milwaukee; 17 Division of Pediatric Surgery, Riley Children’s Hospital, Indiana University School of Medicine, Indianapolis; 18 Division of Pediatric Surgery, American Family Children’s Hospital, University of Wisconsin, Madison; 19 Division of Pediatric Surgery, Comer Children’s Hospital, University of Chicago Medicine and Biological Sciences, Chicago, Illinois

## Abstract

**Importance:**

The ability of computed tomography (CT) to distinguish between benign congenital lung malformations and malignant cystic pleuropulmonary blastomas (PPBs) is unclear.

**Objective:**

To assess whether chest CT can detect malignant tumors among postnatally detected lung lesions in children.

**Design, Setting, and Participants:**

This retrospective multicenter case-control study used a consortium database of 521 pathologically confirmed primary lung lesions from January 1, 2009, through December 31, 2015, to assess diagnostic accuracy. Preoperative CT scans of children with cystic PPB (cases) were selected and age-matched with CT scans from patients with postnatally detected congenital lung malformations (controls). Statistical analysis was performed from January 18 to September 6, 2020. Preoperative CT scans were interpreted independently by 9 experienced pediatric radiologists in a blinded fashion and analyzed from January 24, 2019, to September 6, 2020.

**Main Outcomes and Measures:**

Accuracy, sensitivity, and specificity of CT in correctly identifying children with malignant tumors.

**Results:**

Among 477 CT scans identified (282 boys [59%]; median age at CT, 3.6 months [IQR, 1.2-7.2 months]; median age at resection, 6.9 months [IQR, 4.2-12.8 months]), 40 cases were extensively reviewed; 9 cases (23%) had pathologically confirmed cystic PPB. The median age at CT was 7.3 months (IQR, 2.9-22.4 months), and median age at resection was 8.7 months (IQR, 5.0-24.4 months). The sensitivity of CT for detecting PPB was 58%, and the specificity was 83%. High suspicion for malignancy correlated with PPB pathology (odds ratio, 13.5; 95% CI, 2.7-67.3; *P* = .002). There was poor interrater reliability (κ = 0.36 [range, 0.06-0.64]; *P* < .001) and no significant difference in specific imaging characteristics between PPB and benign cystic lesions. The overall accuracy rate for distinguishing benign vs malignant lesions was 81%.

**Conclusions and Relevance:**

This study suggests that chest CT, the current criterion standard imaging modality to assess the lung parenchyma, may not accurately and reliably distinguish PPB from benign congenital lung malformations in children. In any cystic lung lesion without a prenatal diagnosis, operative management to confirm pathologic diagnosis is warranted.

## Key Points

**Question:**

Can chest computed tomography be used to detect malignant tumors in children with cystic lung lesions?

**Findings:**

In this multi-institutional case-control study of 40 children with cystic lung lesions, the sensitivity for detecting pleuropulmonary blastoma was 58%, and the specificity was 83%. Although high suspicion for malignancy was significantly correlated with malignant tumors, the diagnostic accuracy was 81%, and agreement among radiologists was poor.

**Meaning:**

This study suggests that computed tomography may not accurately and reliably identify pleuropulmonary blastoma; operative management is therefore recommended to confirm pathologic diagnosis in postnatally detected cystic lesions.

## Teaser

This case-control study assesses whether chest computed tomography (CT) can detect malignant tumors among postnatally detected lung lesions in children.

## Introduction

Congenital lung malformations (CLMs) are a group of benign pulmonary anomalies that include bronchogenic cysts, bronchopulmonary sequestration (BPS), congenital lobar emphysema, and congenital pulmonary airway malformations (CPAMs).^1,2,3^ The incidence of these lung lesions has increased during the past 20 years owing to the more widespread use of imaging, and some studies suggest that these lung lesions occur in up to 1 in 2000 children.^4,5^ Congenital lung malformations are known to be associated with a wide spectrum of disease, ranging from in utero hydrops and respiratory distress at birth to pneumothorax and recurrent pneumonia during the first several years of life.^6,7^ Although symptomatic lesions are ubiquitiously managed by surgical resection,^8,9,10,11^ asymptomatic lesions during early infancy have an unclear natural history. Accordingly, some clinicians have espoused nonoperative management strategies for asymptomatic CLMs and recommend delayed surgical resection only if clinical symptoms arise.^12,13,14^

Although malignant transformation of benign CLMs is thought to be uncommon,^15^ a recent multicenter study^16^ confirmed that approximately 10% of cystic lung lesions that were initially diagnosed in the postnatal period may harbor cystic (type I) pleuropulmonary blastoma (PPB), a malignant and potentially lethal lung tumor associated with the *DICER1* (OMIM 601200) variant.^17^ Many PPBs have a similar appearance to macrocystic CPAMs on computed tomography (CT) scans, the current criterion standard imaging modality to assess the lung parenchyma in children, based on several retrospective reviews.^15,18,19,20,21,22,23^ The reliability of CT in cases of PPB remains unknown.^24^

Given ongoing concerns about misdiagnosing PPB as a benign macrocytic CPAM, we used a multi-institutional consortium registry to formally evaluate the CT characteristics of cystic PPB and benign CLMs. The primary aim of this study was to assess whether a cohort of board-certified pediatric radiologists could accurately discern PPB from CLMs when evaluating the same CT images. We hypothesized that experienced radiologists with an increased awareness of PPB could reliably diagnose most PPBs and CPAMs with acceptable agreement.

## Methods

### Study Design, Setting, Participants

A central reliance agreement was approved by the institutional review boards of all institutions associated with the Midwest Pediatric Surgery Consortium, a group of 11 US tertiary care children’s hospitals located in 7 contiguous states serving an estimated total population of 58 million people (Children’s Mercy Hospital, Kansas City, Missouri; Children’s Wisconsin, Milwaukee; St Louis Children’s Hospital, St Louis, Missouri; Norton Children’s Hospital, Louisville, Kentucky; Cincinnati Children’s Hospital, Cincinnati, Ohio; Ann & Robert H. Lurie Children’s Hospital, Chicago, Illinois; Nationwide Children’s Hospital, Columbus, Ohio; C. S. Mott & Von Voigtlander Children’s Hospital, Ann Arbor, Michigan; Riley Children’s Hospital, Indianapolis, Indiana; Comer Children’s Hospital, Chicago, Illinois; and American Family Children’s Hospital, Madison, Wisconsin).^25^ Informed consent was waived owing to minimal risk to participants. Original CT reports (denoted as R0) and pathology records were queried from an operative registry of 521 primary lung lesions resected between January 1, 2009, and December 31, 2015, and detailed elsewhere.^26,27^ All preoperative chest CT scans were performed with intravenous contrast, using a variety of CT scanners with a minimum of 16 slices.^28^ This study followed the Strengthening the Reporting of Observational Studies in Epidemiology (STROBE) reporting guideline.

### Eligibility Criteria

The study cohort generated for detailed CT analysis is shown in eFigure 1 in Supplement 1. All children in the registry with evaluable preoperative chest CT images and an original imaging report were screened. We excluded prenatally diagnosed lesions given the known association of PPB with an initial diagnosis in the postnatal period.^6,16,20,29,30^ Computed tomography scans from pathologically confirmed cystic PPBs (cases) were then individually age-matched with CT scans from pathologically confirmed benign CLMs (controls) at a target ratio of 1:3. We deliberately enriched for macrocystic CPAMs when possible and selected against BPS and CPAM with systemic feeding vessels, given the known association between systemic feeding vessels and benign disease.^18^ Images were stripped of patient identifiers and uploaded to the InteleViewer picture achiving and communication system (Intelerad Medical Systems Inc) for radiologic interpretation.

### Variables

Board-certified pediatric radiologists were recruited to review these images in an independent and blinded fashion from January 24, 2019, to September 6, 2020. Each radiologist had no knowledge of the original CT diagnosis, pathologic diagnosis, and number or proportion of PPB lesions in the study cohort. In addition to recording data on lesion characteristics and a final radiologic diagnosis, each study radiologist provided a confidence level in (1) the radiologic diagnosis and (2) overall suspicion of malignancy using 5-point Likert scales (range, 1-5, where 1 = very likely benign and 5 = almost certainly malignant). Data were stored in a centralized REDCap database (Research Electronic Data Capture, version 8.1.20; Vanderbilt University). Outcomes of interest were diagnostic accuracy, interrater reliability, correlation of radiologist suspicion with pathology diagnosis, and radiologic characterization of cystic PPB lesions.

### Statistical Analysis

Statistical analysis was performed from January 18 to September 6, 2020, using standard parametric methods for continuous and categorical variables. Binary logistic regression models were performed as appropriate using Stata, version 16.1 (StataCorp LLC) or R Studio, version 3.6.1 (R Group for Statistical Computing). All *P* values were from 2-sided tests and results were deemed statistically significant at *P* < .05. Positive predictive values (PPVs) and negative predictive values (NPVs) were calculated based on PPB prevalence estimates as described elsewhere.^16,23^ Interrater reliability was assessed using the Cohen κ statistic and intraclass correlation coefficient with 2-way mixed-effects modeling. The following levels of interrater agreement were used for interpretation: lower than 0.50 = poor, 0.50 to 0.74 = moderate, 0.75 to 0.90 = good, and higher than 0.90 = excellent.^31^

## Results

### Patient Characteristics

eTable 1 in Supplement 1 shows the baseline characteristics of the 477 registry patients (282 boys [59%]; median age at CT, 3.6 months [IQR, 1.2-7.2 months]; median age at resection, 6.9 months [IQR, 4.2-12.8 months]) with at least 1 preoperative CT scan. Pathologic diagnoses included BPS (158 patients [33%]), bronchial atresia (13 patients [3%]), bronchogenic cyst (31 patients [7%]), congenital lobar emphysema (50 patients [11%]), CPAM (210 patients [44%]), and cystic PPB (11 patients [2%]). No malignant tumors were identified in the 314 lesions (66%) that were initially detected prenatally.

Forty age-matched children (8%) with primary lung lesions were selected for detailed radiologic review (eTable 1 in Supplement 1). There were 26 right-sided lesions (65%) and 15 left-sided lesions (38%). The most common anatomic locations were right lower lobe (12 [30%]), right upper lobe (9 [23%]), and left lower lobe (8 [20%]). There were 20 CPAMs (50%) and 9 (23%) pathologically confirmed cystic PPBs. In accordance with the study design, the cohort included significantly fewer lung malformations associated with a systemic feeding vessel when compared with the entire registry cohort. Adequacy of age matching between benign and malignant cases was demonstrated by the lack of significant differences in median age at CT (benign, 7.3 months [IQR, 2.1-22.3 months] vs malignant, 8.2 months [IQR, 4.0-70.1 months]; *P* = .75) and in median age at resection (benign, 8.7 months [IQR, 5.0-24.4 months] vs malignant, 10.1 months [IQR, 4.2-71.2 months]; *P* = .83).

### Diagnostic Accuracy

#### Original Radiologist

Data on the original diagnostic accuracy of chest CT for the 477 lesions are presented in eTable 2 in Supplement 1. The sensitivity for detecting a CPAM was 87% (95% CI, 81%-91%) and the specificity was 80% (95% CI, 75%-85%). The PPV for CPAM was 78% (95% CI, 73%-82%) and the NPV was 88% (95% CI, 84%-92%). For the other benign lung malformations, the sensitivity of CT was lower (range, 41%-74%), but there was higher specificity (range, 96%-100%). The sensitivity for detecting a malignant primary lesion was 33% (95% CI, 12%-62%) and the specificity was 99% (95% CI, 98%-100%). Based on the overall low prevalence of malignant lung lesions in the registry, the PPV for a malignant primary lesion was 50% (95% CI, 25%-76%) and the NPV was 98% (95% CI, 95%-98%).

#### Study Radiologists

There were 9 study radiologists (denoted as radiologists R1-R9) representing 6 different consortium hospitals. They collectively completed a total of 346 of 360 interpretations (96%). Fourteen of 360 studies (4%) could not be evaluated by 2 study radiologists (R3 and R4) owing to hardware or software compatibility issues. In addition to board certification among all reviewers, experience in pediatric chest CT scan interpretation was confirmed by number of years after pediatric radiology fellowship training (median, 9 years; range, 4-31 years). The median estimated number of lung lesion CT scans reviewed annually per study radiologist was 10 (range, 2-15).

Table 1 compares the CT diagnosis for all images based on the original interpretation and the study radiologist’s interpretation. The most common diagnosis in both groups was CPAM; study radiologists were significantly less likely to interpret a study as CPAM compared with those analyzed by the original interpretation (149 of 346 [43%] vs 29 of 40 [73%]; *P* = .009). Conversely, the percentage of studies for which the study radiologists believed that PPB was the most likely diagnosis was significantly increased when compared with those interpreted by the original radiologist (88 of 346 [25%] vs 3 of 40 [8%]; *P* = .008).

**Table 1.** Computed Tomography of 40 Lung Lesions: Original Interpretation vs Study Interpretation

Characteristic	No. (%)		*P* value
	Original interpretation	Study interpretation^a^	
No. of reads	40	346	NA
Computed tomography diagnosis			
BPS	0	28 (8)	.10
Bronchial atresia	0	12 (4)	.62
Bronchogenic cyst	2 (5)	6 (2)	.20
CLE	2 (5)	25 (7)	>.99
CPAM	29 (73)	149 (43)	.009^b^
CPAM with systemic feeding vessel	1 (3)	21 (6)	.71
Infectious or pneumatoceles	2 (5)	3 (1)	.09
Miscellaneous benign	2 (5)	11 (3)	.63
Other malignant tumor^c^	0	4 (1)	>.99
PPB	3 (8)	88 (25)	.008^b^

Abbreviations: BPS, bronchopulmonary sequestration; CLE, congenital lobar emphysema; CPAM, congenital pulmonary airway malformation; NA, not applicable; PPB, pleuropulmonary blastoma.

^a^ Nine radiologists independently reviewed the same 40 studies (3.9% noncompletion rate).

^b^ *P* < .05 (Fisher exact test).

^c^ Includes bronchoalveolar carcinoma and pulmonary sarcoma.

Among the study radiologists, the overall diagnostic accuracy, defined as the proportion of true positives and true negatives, for all lung lesions was 81%. Study radiologists increased the sensitivity for detecting a malignant primary lesion to 58% but had reduced specificity (83%) when compared with the original CT interpretation (eTable 2 in Supplement 1). Based on the estimated disease prevalence of PPB among postnatally diagnosed lung lesions,^16^ the calculated PPV was 24% and the NPV was 95%. The sensitivity of the study radiologists for detecting a CPAM was 56% and the specificity was 70%.

**Figure 1.**
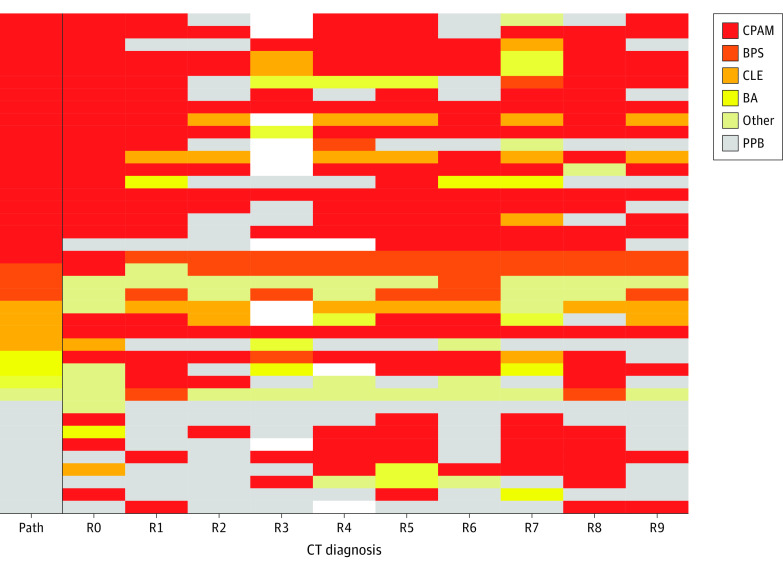
Heatmap Illustrating the Variability in Computed Tomography (CT) Diagnosis Among 40 Lung Lesions The correct diagnosis (pathology) is indicated in the far-left column. Nonevaluable studies are shown in white. BA indicates bronchial atresia; BPS, bronchopulmonary sequestration; CLE, congenital lobar emphysema; CPAM, congenital pulmonary airway malformation; Path, pathology; PPB, pleuropulmonary blastoma; R0, original interpretation; and R1-R9, study interpretation by 1 of 9 radiologists.

**Figure 2.**
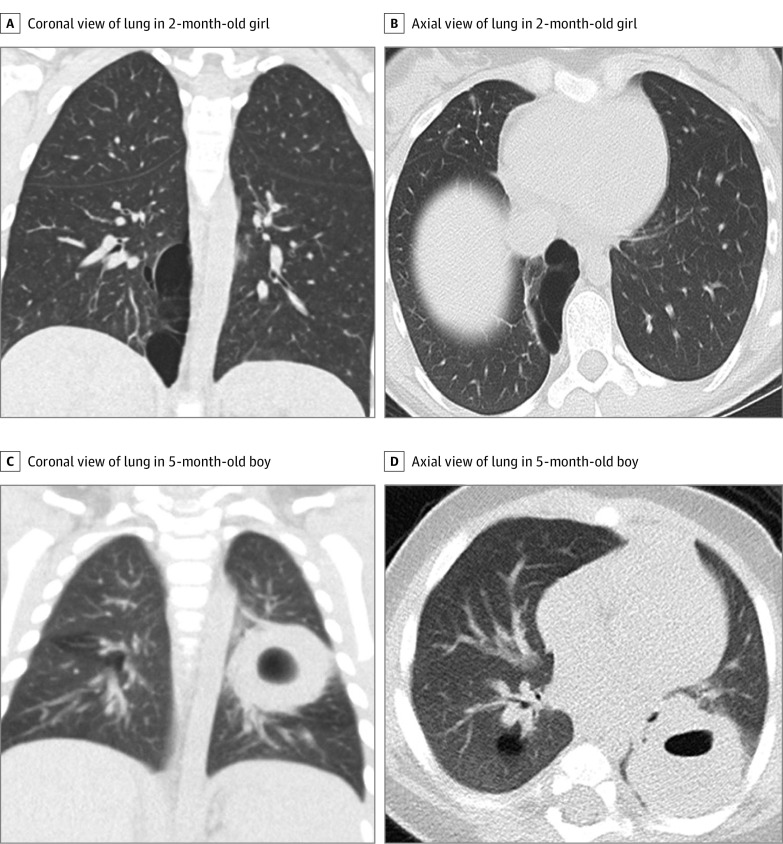
Examples of Computed Tomography Images Reviewed by Study Radiologists Representative coronal (A) and axial (B) lung window images in a 2-month-old girl show a 5.5 × 2.6 × 8.9-cm cystic lung lesion located in the medial right lower lobe with thin internal septations. The pathologic diagnosis was pleuropulmonary blastoma (PPB) but was interpreted by 6 study radiologists as a benign macrocystic congenital pulmonary airway malformation (CPAM). Representative coronal (C) and axial (D) lung window images in a 5-month-old boy with a posterior left lower lobe 2.9 × 3.5 × 2.7-cm solid lung lesion with an internal cyst. The pathologic diagnosis was CPAM but was diagnosed by 6 study radiologists as PPB.

The variability in CT diagnosis is depicted by the heatmap shown in Figure 1. Although many lesions were correctly diagnosed by CT, the heatmap revealed a discordance with pathologic diagnosis in many cases, and there was lack of consensus in the radiologic diagnosis across the spectrum of primary lung lesions. Congenital pulmonary airway malformations, congenital lobar emphysema, bronchogenic cysts, and bronchial atresias were all misclassified as malignant tumors and other types of benign lesions (Figure 2). For PPB, the false-positive rate was 18% (49 of 267 reads). Bronchopulmonary sequestration was the only CLM pathology that was not diagnosed as a PPB by a radiologist. The interrater reliability score as measured by the intraclass correlation was 0.31 (95% CI, 0.18-0.49) and as measured by the combined κ statistic was 0.36 (range, 0.06-0.64). Both values indicate poor agreement with regard to whether a given lesion was benign vs malignant.

To assess whether there might be an association between radiology experience and diagnostic accuracy in the detection of PPB, we calculated the sensitivity and specificity for each study radiologist and correlated these findings with their reported total number of lung lesion chest CT scans reviewed since completion of fellowship training (eFigure 2 in Supplement 1). Although there were no obvious differences in specificity rates associated with radiologist experience, less-experienced radiologists tended to have lower sensitivity rates for detecting malignant lesions compared with their more experienced colleagues.

### Malignancy Risk Assessment

Based on a 5-point Likert scale (range, 1-5, where 1 = very likely benign and 5 = almost certainly malignant), the mean (SD) overall diagnostic confidence score was 3.9 (0.9), signifying a moderate-to-high level of diagnostic certainty by CT. The mean (SD) level of suspicion of malignancy was 1.9 (1.2), indicating a relatively low level of suspicion of malignancy within the study cohort. To assess the association between CT and pathologic diagnosis, binary logistic regressions were performed based on Likert scales (Table 2). This analysis revealed a significant, 6-fold increased risk for malignant tumors based on a positive radiologist impression for PPB (odds ratio, 6.4; 95% CI, 2.8-14.5; *P* = .01). Moreover, each additional 1 point on the Likert scale correlated with an increased risk for a PPB diagnosis (suspicion level 2: odds ratio, 2.0; 95% CI, 1.2-3.3; and suspicion level 5: odds ratio, 13.5; 95% CI, 2.7-67.3).

**Table 2.** Correlation of Study Radiologists’ CT Diagnosis and Suspicion With Pathology Diagnosis

Characteristic	Odds ratio (95% CI)	*P* value^a^
CT diagnosis of PPB	6.4 (2.8-14.5)	<.001
Suspicion level^b^		
2	2.0 (1.2-3.3)	.006
3	3.7 (1.8-8.0)	.001
4	8.4 (2.6-27.1)	<.001
5	13.5 (2.7-67.3)	.002

Abbreviations: CT, computed tomography; PPB, pleuropulmonary blastoma.

^a^ *P* < .05 indicates significance.

^b^ Compared with a score of 1 (lowest suspicion) on a 5-point Likert scale.

### CT Characteristics of PPB

To identify lesion characteristics on CT scans that might be correlated with PPB, specific imaging findings were systematically reviewed (Table 3). Bivariate analysis showed that left upper lobe lesions (benign, 4 of 31 [13%]; and malignant, 5 of 9 [56%]; *P* = .03) and macrocystic lesions (benign, 20 of 31 [65%]; and malignant, 9 of 9 [100%]; *P* = .04) were significantly associated with a pathologic diagnosis of PPB. No CT characteristic was found to be significant for benign or malignant disease in multivariable logistic regression.

**Table 3.** Computed Tomography Characteristics of Benign vs Malignant Lesions

Characteristic	No. (%)		*P* value
	Benign (n = 31)	Malignant (n = 9)	
Anatomic location			
Right			
Upper lobe	8 (26)	3 (33)	>.99
Middle lobe	4 (13)	0	.56
Lower lobe	12 (39)	1 (11)	.13
Left			
Upper lobe	4 (13)	5 (56)	.03^a^
Lower lobe	8 (26)	2 (22)	>.99
Laterality			
Right	20 (65)	3 (33)	.13
Left	9 (29)	5 (56)	.23
Bilateral	2 (7)	1 (11)	.55
Maximum cyst size, mean (SD), cm	6.3 (2.8)	6.9 (3.4)	.10
Solid portion	11 (36)	5 (56)	.44
Cyst characteristics			
Macrocystic (>1 cm)	20 (65)	9 (100)	.04^a^
Microcystic (<1 cm)	2 (7)	0	>.99
Midline shift	14 (45)	4 (44)	>.99
Pneumothorax	0	1 (11)	.23
Pleural effusion	3 (10)	2 (22)	.31
Septations	15 (48)	8 (89)	.05
Chest wall invasion	1 (3)	0	>.99
Mediastinal lymph nodes	3 (10)	1 (11)	>.99

^a^ *P* < .05 (Fisher exact or *t* test).

## Discussion

The ability of cross-sectional imaging to discern between malignant and benign cystic lung lesions is of critical importance in guiding preoperative counseling discussions with families and in deciding whether to proceed with operative vs nonoperative management strategies.^29^ In this study, CT scans from young children (median age, 7-8 months) with either a pathologically confirmed benign CLM or confirmed malignant PPB were reviewed independently in a blinded fashion by a cohort of trained pediatric radiologists. There are several important findings. First, CT scans have limited diagnostic accuracy in terms of distinguishing between benign and malignant cystic lung disease. Among our study radiologists, the sensitivity for detecting PPB was 58% and the overall specificity was 83%. Less-experienced radiologists tended to have lower sensitivity rates for detecting malignant lesions compared with more-experienced radiologists, and the overall diagnostic accuracy was 81%. Based on reports from the original radiologist interpretation, the sensitivity for detecting PPB lesions was 33% and the specificity was 99%. Consistent with the difficulties in accurately diagnosing CLM lesions in general, these data validate the handful of radiology report–based studies that document a high rate of misdiagnosis of cystic PPB lesions as benign disease.^20,32,33,34^

Although the enhanced sensitivity observed by the study radiologists as a group may be a function of their increased experience with this imaging modality for young children, the change in study diagnostic accuracy may also be secondary to a Hawthorne effect,^35^ in which an increased awareness of the study aims influenced a given radiologist’s threshold to diagnosis a cystic lesion as malignant. The presence of a Hawthorne effect in our study therefore represents the best-case scenario for detecting PPB among a group of benign lung lesions. Regardless, the difficulty in establishing a CT diagnosis, which is further demonstrated by minimal interobserver agreement among these reviewers, highlights the current limitations of chest CT in terms of distinguishing benign and malignant cystic lung lesions in children. Other investigators have also observed that cystic PPB may be indistinguishable from CPAM by CT.^20,21,22,36^ Although magnetic resonance imaging may play a diagnostic role in a few cases, this imaging modality usually provides suboptimal visualization of the lung parenchyma owing to low magnetic resonance signal from proton-poor lung tissue.^37^

A second major finding from our study was the lack of specific imaging characteristics on CT scans that help to reliably discern PPB lesions from macrocystic CPAMs. Genetic and histologic investigations have confirmed distinct pathogenic mechanisms between these 2 pulmonary diseases.^21,38,39^ Less than 5% of PPBs are detected prenatally.^18,20,40,41^ The only radiologic feature that is thought to be pathognomic for benign disease is the presence of a systemic feeding vessel,^16,18^ and our study radiologists correctly excluded malignancy in their review of all confirmed BPS lesions. The significance of other image-based characteristics in identifying PPB is more controversial.^20^ Prior work based on radiology reports has suggested that certain features, particularly multifocalilty or biliateral lesions, are more consistent with malignant disease.^18,42^ However, our study, which was restricted to postnatally diagnosed lesions, revealed that bilateral disease, midline shift, pneumothorax, and pleural effusion are uncommon and not specific for malignant pathology.^43^ Based on data from the International Pleuropulmonary Blastoma Registry (IPBR), the incidence of bilateral involvement in cystic PPB was only 10% to 20%, multifocal disease ranged from 5% to 40%, and pneumothorax was only 30%.^18,22,44^ Therefore, CT scans of most PPB lesions would not show these high-risk characteristics. Data from IPBR-based studies have suggested that cystic PPB is associated with larger cyst size,^18,44^ but our analysis did not find that overall maximum cyst size was significantly larger in PPB lesions when compared with CPAMs enriched with macrocystic disease. Contrary to previous studies that have described a right-sided predilection for PPB,^20,43,45^ we found that left upper lobe involvement was significantly more common in PPB.

In spite of the inherent limitations of chest CT in terms of accurately detecting some PPB lesions, our data did reveal that suspected PPB cases correlated with an increased likelihood for a malignant pathologic diagnosis. In the most highly suspicious scans, there was a more than 13-fold increased likelihood of PPB. These results are concordant with previous work suggesting a relatively low false-positive rate of CT for a PPB diagnosis.^16,18^ However, when adjusted for the estimated diseases prevalence of PPB, this translated to a PPV of only 24%, which is much lower than rates from the original radiology reports and those reported elsewhere.^18^ One possible explanation for the lower predictive values might be the absence of clinical data available at the time of interpretation. Family history, *DICER1* variant status, and clinical symptoms have all been shown to correlate with the diagnosis of cystic PPB. Clinical symptoms of PPB can be insidious, and some children with PPB are asymptomatic during the early stages.^16,18,20,33^ It has also been shown that heterozygous germline *DICER1* variants, which are inherited in an autosomal dominant fashion, have an estimated penetrance of only 10% to 15%.^46,47^ Furthermore, there is a nontrivial rate of sporadic PPB cases in children who have no positive family history of *DICER1*-related cancers.^22^ Some studies indicate that one-third of histologically confirmed cystic PPBs are not associated with a *DICER1* variant.^18,44^ Taken together, these data would imply that reliable preoperative exclusion of a malignant tumor on the basis of reassuring radiologic findings in combination with a negative test result for *DICER1* variants is not possible at the present time for any child with a postnatally detected macrocystic lung lesion.

Although the estimated NPV for a malignant lesion was 95%, our data reveal that many PPB lesions may not be appropriately recognized when suggested management algorithms are followed.^18^ We therefore advocate for operative management to confirm pathologic diagnosis in any cystic lung lesion without an antenatal diagnosis and do not recommend routine expectant management.^48,49,50^ Achieving total resection at an early age is associated with positive outcomes in the management of PPB.^33,40,44,51,52^ Surgical resection of cystic PPB managed at a median age of diagnosis at 8 months is associated with a 5-year survival rate of 91%. Most PPB deaths are associated with progression to type II or III disease.^21,44^ Pleuropulmonary blastoma with solid components, which have an older median age of diagnosis, are associated with bone, brain, and liver metastasis; require adjuvant therapy in addition to surgical resection; and have significantly lower 5-year survival rates (51%-73%).^44^

### Limitations

This study has some limitations. Our study cohort of primary lung lesions was kept intentionally small, thereby enabling a group of radiologists to carefully review 40 studies. However, as is inherent to research on rare diseases, the relatively low numbers of PPB lesions evaluated may have resulted in a lack of power to detect rare but statistically significant differences in imaging characteristics associated with malignant tumors. Larger prospective studies to assess the role of specific imaging features in pathologic diagnosis would likely require multidisciplinary, international collaborative efforts through the IPBR or elsewhere, perhaps in combination with machine learning algorithms.^53^ There may have also been differences in CT image acquisition and image quality that hampered the ability to interpret some of these images with optimal diagnostic accuracy. Additionally, in addition to concerns about the Hawthorne effect, the external validity of our findings may be questioned when applied to other pediatric hospital settings where the imaging technology and radiology expertise required to interpret these CT scans may differ.

## Conclusions

Based on this regional multi-institutional case-control study, our hypothesis that CT can reliably discern between cystic PPBs and benign CLMs without a systemic feeding vessel should be rejected. Consequently, operative management to confirm pathologic diagnosis is warranted in any cystic lung lesion without prenatal detection.

## Notes

Supplement 1. **eTable 1.** Baseline Characteristics of 477 Children With Primary Lung Lesions Detected by Chest CT **eTable 2.** Diagnostic Accuracy Based on Original Radiologist Interpretation of 477 Primary Lung Lesions **eFigure 1.** Flow Diagram of Inclusion and Exclusion Criteria Used to Generate Study Cohort of Children Undergoing Blinded Preoperative Chest Computed Tomography (CT) Review for a Pathology Confirmed Lung Lesion **eFigure 2.** Scatter Plot Showing Association Between Radiologist Experience (x-axis), as Measured by the Estimated Number of Lung Lesion Chest Computed Tomography (CT) Scans Reviewed, and Sensitivity/Specificity for Identifying a Malignant Lesion (y-axis)

Supplement 2.
Nonauthor Collaborators
