# Supplementary material for: Accuracy of Chest Computed Tomography in Distinguishing Cystic Pleuropulmonary Blastoma From Benign Congenital Lung Malformations in Children
Source: JAMA Netw Open. 2022 Jun 30;5(6):e2219814. doi: 10.1001/jamanetworkopen.2022.19814 (PMC9247735; doi:10.1001/jamanetworkopen.2022.19814)
Supplement: Supplement 1. — eTable 1. Baseline Characteristics of 477 Children With Primary Lung Lesions Detected by Chest CT eTable 2. Diagnostic Accuracy Based on Original Radiologist Interpretation of 477 Primary Lung Lesions eFigure 1. Flow Diagram of Inclusion and Exclusion Criteria Used to Generate Study Cohort of Children Undergoing Blinded Preoperative Chest Computed Tomography (CT) Review for a Pathology Confirmed Lung Lesion eFigure 2. Scatter Plot Showing Association Between Radiologist Experience (x-axis), as Measured by the Estimated Number of Lung Lesion Chest Computed Tomography (CT) Scans Reviewed, and Sensitivity/Specificity for Identifying a Malignant Lesion (y-axis) [file jamanetwopen-e2219814-s001.pdf]

Supplementary Online Content

Engwall-Gill AJ, Chan SS, Boyd KP, et al; Midwest Pediatric Surgery Consortium. Accuracy of chest computed tomography in distinguishing cystic pleuropulmonary blastoma from benign congenital lung malformations in children. *JAMA Netw Open.* 2022;5(6):2219814. doi:10.1001/jamanetworkopen.2022.19814

eTable 1. Baseline Characteristics of 477 Children With Primary Lung Lesions Detected by Chest CT
eTable 2. Diagnostic Accuracy Based on Original Radiologist Interpretation of 477 Primary Lung Lesions
eFigure 1. Flow Diagram of Inclusion and Exclusion Criteria Used to Generate Study Cohort of Children Undergoing Blinded Preoperative Chest Computed Tomography (CT) Review for a Pathology Confirmed Lung Lesion
eFigure 2. Scatter Plot Showing Association Between Radiologist Experience (x-axis), as Measured by the Estimated Number of Lung Lesion Chest Computed Tomography (CT) Scans Reviewed, and Sensitivity/Specificity for Identifying a Malignant Lesion (y-axis)

This supplementary material has been provided by the authors to give readers additional information about their work.

© 2022 Engwall-Gill AJ et al. *JAMA Network Open.*

eTable 1. Baseline Characteristics of 477 Children With Primary Lung Lesions Detected by Chest CT

Variable	*All lesions (n=477)*	*Case-control group (n=40)*	*Excluded (n=437)*	*p value*
Male gender, n (%)	282 (59.2)	27 (67.5)	255 (58.4)	0.31
Age at preoperative CT, median (IQR), months	3.6 (1.2-7.2)	7.3 (2.9-22.4)	3.2 (1.1-6.7)	0.75
Age at resection, median (IQR), months	6.9 (4.2-12.8)	8.7 (5.0-24.4)	6.8 (4.0-11.4)	0.83
Prenatal diagnosis, n (%)	314 (65.8)	0 (0.0)	314 (71.9)	<0.01*
Bilateral disease, n (%)	2 (0.4)	0 (0.0)	2 (0.5)	1.00
Anatomic location, n (%)				
Right upper lobe	58 (12.2)	9 (22.5)	49 (11.2)	0.04*
Right middle lobe	40 (8.4)	5 (12.5)	35 (8.0)	0.36
Right lower lobe	143 (29.9)	12 (30.0)	131 (30.0)	1.00
Left upper lobe	66 (13.9)	7 (17.5)	59 (13.5)	0.47
Left lower lobe	138 (29.1)	8 (20.0)	130 (29.8)	0.27
Extralobar	62 (13.1)	3 (7.5)	59 (13.5)	0.46
Pathologic diagnosis, n (%)				
BPS	158 (33.5)	4 (10.0)	154 (35.9)	<0.01*
Bronchial atresia	13 (2.8)	1 (2.5)	12 (2.8)	1.00
Bronchogenic cyst	31 (6.6)	2 (5.0)	29 (6.7)	1.00
CLE	50 (10.7)	4 (10.0)	46 (10.7)	1.00
CPAM	210 (44.9)	20 (50.0)	190 (44.2)	0.51
CPAM with systemic feeding vessel	44 (9.4)	0 (0.0)	44 (10.2)	0.04*
Malignancy, other	4 (0.8)	0 (0.0)	4 (0.9)	1.00
PPB, type 1/cystic	11 (2.3)	9 (22.5)	2 (0.5)	<0.01*

© 2022 Engwall-Gill AJ et al. *JAMA Network Open.*

Abbreviations: CT, computed tomography; IQR, interquartile range; BPS, bronchopulmonary sequestration; CLE, congenital lobar emphysema; CPAM, congenital pulmonary airway malformation; PPB, pleuropulmonary blastoma
* $p<0.05$ (Fisher's exact test) between case-control and excluded patients

© 2022 Engwall-Gill AJ et al. *JAMA Network Open.*

eTable 2. Diagnostic Accuracy Based on Original Radiologist Interpretation of 477 Primary Lung Lesions

Pathology	*Sensitivity*	*Specificity*	*Positive predictive value*	*Negative predictive value*
BPS, % (95% CI)	61.1 (53.1-68.8)	97.5 (95.1-98.9)	92.3 (85.7-96.0)	83.7 (80.8-86.2)
Bronchogenic cyst, % (95% CI)	51.6 (33.1-69.9)	99.8 (98.8-100.0)	94.1 (68.7-99.2)	96.7 (95.4-97.7)
Congenital lobar emphysema, % (95% CI)	74.0 (59.7-85.4)	97.7 (95.7-98.9)	78.7 (66.2-87.5)	97.0 (95.3-98.1)
CPAM, % (95% CI)	86.7 (81.3-91.0)	80.2 (74.9-84.8)	77.5 (72.9-81.5)	88.4 (84.3-91.6)
CPAM with feeder, % (95% CI)	40.9 (26.3-56.8)	96.1 (93.8-97.7)	51.4 (37.1-65.5)	94.1 (92.6-95.3)
Malignancy, % (95% CI)	33.3 (11.8-61.6)	98.9 (97.5-99.6)	50.0 (24.5-75.5)	97.8 (94.8-98.2)

Abbreviations: CT, computed tomography; BPS, bronchopulmonary sequestration; CPAM, congenital pulmonary airway malformation; CI, confidence interval

© 2022 Engwall-Gill AJ et al. *JAMA Network Open.*

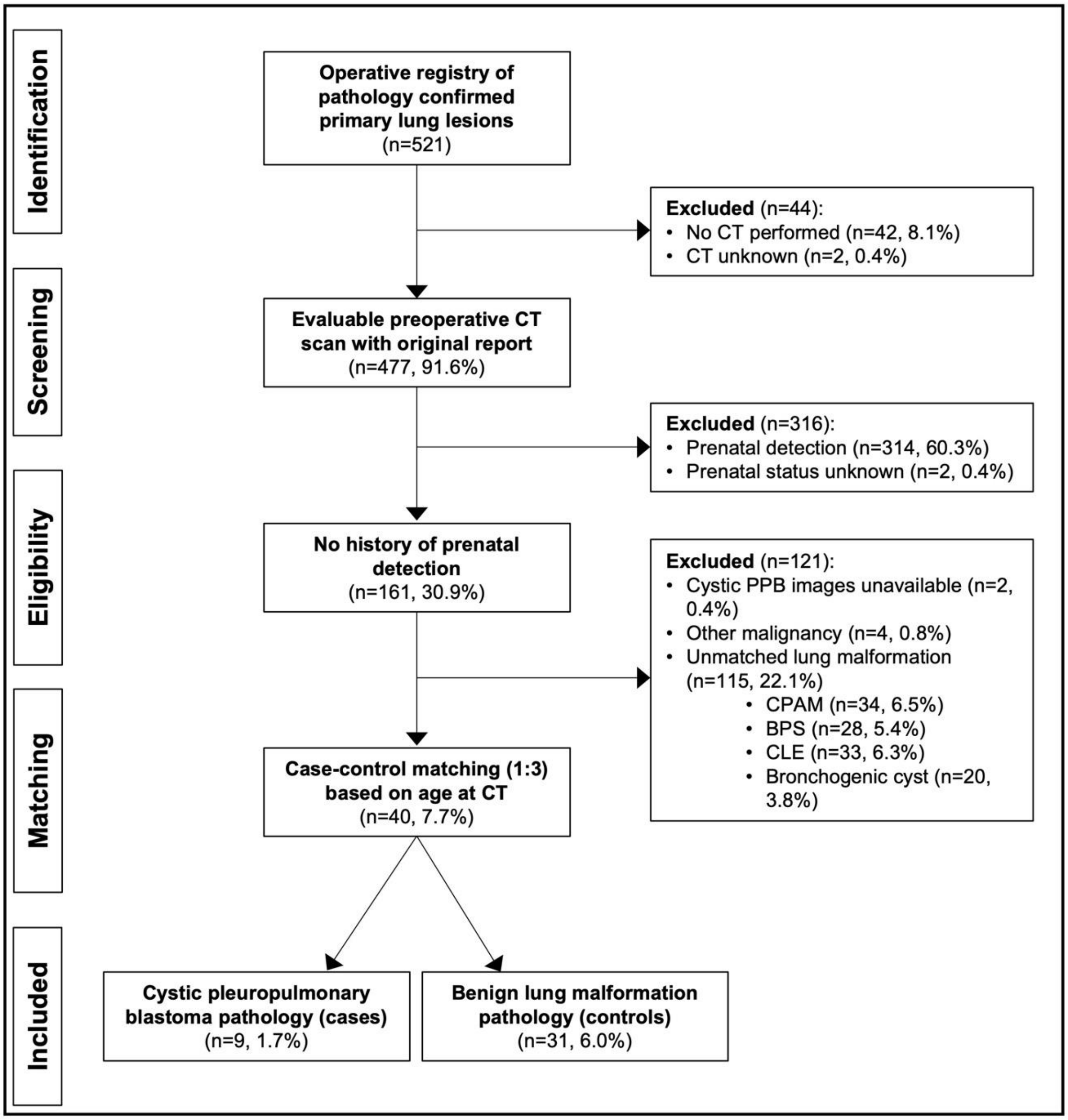

eFigure 1. Flow Diagram of Inclusion and Exclusion Criteria Used to Generate Study Cohort of Children Undergoing Blinded Preoperative Chest Computed Tomography (CT) Review for a Pathology Confirmed Lung Lesion. Prenatally detected lesions were excluded. PPB, pleuropulmonary blastoma; CPAM, congenital pulmonary airway malformation; BPS, bronchopulmonary sequestration; CLE, congenital lobar emphysema

© 2022 Engwall-Gill AJ et al. *JAMA Network Open.*

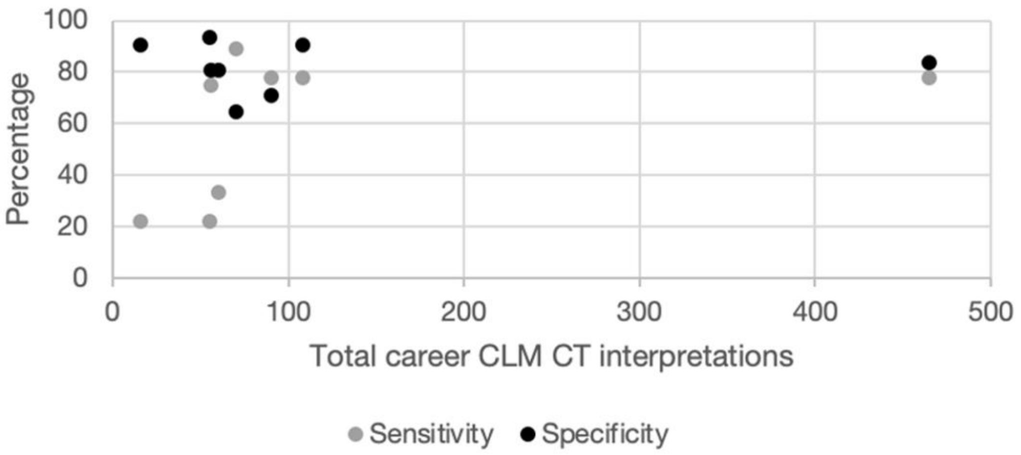

eFigure 2. Scatter Plot Showing Association Between Radiologist Experience (x-axis), as Measured by the Estimated Number of Lung Lesion Chest Computed Tomography (CT) Scans Reviewed, and Sensitivity/Specificity for Identifying a Malignant Lesion (y-axis)

© 2022 Engwall-Gill AJ et al. *JAMA Network Open.*
