# Supplementary material for: Accuracy of Chest Computed Tomography in Distinguishing Cystic Pleuropulmonary Blastoma From Benign Congenital Lung Malformations in Children
Source: JAMA Netw Open. 2022 Jun 30;5(6):e2219814. doi: 10.1001/jamanetworkopen.2022.19814 (PMC9247735; doi:10.1001/jamanetworkopen.2022.19814)
Supplement: Supplement 2. — Nonauthor Collaborators [file jamanetwopen-e2219814-s002.pdf]

Supplemental Online Content: Nonauthor Collaborators
*First name, last name, and suffix (if applicable) are required and will appear in PubMed.

*Group Name(s): Midwest Pediatric Surgery Consortium							
***First Name and Middle Initial(s)**	***Last Name**	***Suffix (eg, Jr, III)**	Academic Degrees	Institution	Location (city, state/province, country)	Role or Contribution, eg, chair, principal investigator	Group (if more than 1 Group listed in the byline) and/or Subgroup (eg, Steering Committee)
Jason	Fraser		MD	Children's Mercy Hospital	Kansas City, MO	Investigator	
Cynthia	Downard		MD	Norton Children's Hospital	Louisville, KY	Investigator	
Cheryl	Adams			Cincinnati Children's Hospital Medical Center	Cincinnati, OH	Investigator	
Thomas	Sato		MD	Children's Wisconsin	Milwaukee, WI	Investigator	
Daniel	von Allmen		MD	Cincinnati Children's Hospital Medical Center	Cincinnati, OH	Investigator	
Jonathan	Kohler		MD	Riley Children's Hospital	Indianapolis, IN	Investigator	
Daniel	Ostlie		MD	American Family Children's Hospital	Madison, WI	Investigator	
Sarah	Fox			C.S. Mott Children's Hospital	Ann Arbor, MI	Project Manager	
